# Interprofessional Training in Virtual Reality for Health Care: Experimental Study on Procedural Knowledge and Willingness to Collaborate

**DOI:** 10.2196/85139

**Published:** 2026-05-27

**Authors:** Melanie Bauer, Matthias J Witti, Matthias Stadler, Anna Weiß, Martin R Fischer, Constanze Richters

**Affiliations:** 1 Institute of Medical Education, LMU University Hospital, LMU Munich, Pettenkoferstr. 8a, Munich, 80336, Germany, 49 89440057204

**Keywords:** virtual reality, immersive, interprofessional education, wound care, cognitive load, procedural knowledge, interprofessional collaboration, medical education, nursing education, pharmacy education

## Abstract

**Background:**

High-quality wound care requires early and effective interprofessional collaboration between medical, nursing, and pharmacy professionals. However, interprofessional education (IPE) in this context remains limited in higher education. Immersive virtual reality (iVR) seems to be a promising IPE tool, enabling a standardized, realistic, and safe learning environment that allows multiple learners from different professions to train together. However, its educational effectiveness likely depends on instructional design that supports learning while managing cognitive demands.

**Objective:**

This study examined whether a newly developed interprofessional iVR wound-care training improves (1) procedural knowledge and (2) willingness to collaborate among medical, nursing, and pharmacy students, and how cognitive load relates to these outcomes.

**Methods:**

A within-subjects design with a pre- and posttest was implemented with 116 students from medicine, nursing, and pharmacy. Students completed 2 iVR sessions (≈25 and 15 min) in interprofessional triads, addressing a pressure ulcer case. The training integrated step-by-step scaffolding for the wound care task and collaboration scripts to guide teamwork. Procedural knowledge and willingness to collaborate were assessed before and after the sessions, and cognitive load was measured after the sessions. Data were analyzed using repeated-measures analysis of covariances and a mediation model to test the preregistered effects.

**Results:**

Procedural knowledge increased significantly from pre- to posttest (*F*_1, 107_=26.19, *P*<.001, *η²*=.08). Cognitive load showed no significant effect on this gain. Willingness to collaborate did not change after the first session (*F*_1, 80_=3.55, *P*=.063, *η²*=.01), and was unaffected by cognitive load. Exploratory analyses showed that willingness to collaborate was significantly higher after the second session (*t*_64_=3.16, *P*=.007, mean difference=0.202). Effects on procedural knowledge and willingness to collaborate did not depend on the learner’s profession.

**Conclusions:**

These findings suggest that the iVR training effectively supported learning by providing a clear structure and managing cognitive demands, enabling students from different professions to acquire procedural knowledge. The absence of cognitive load effects may suggest that the instructional design helped balance task complexity and guidance. The delayed increase in collaboration willingness further suggests that attitudinal change requires sustained, repeated engagement in interprofessional contexts rather than a single exposure. Notably, no profession-related differences emerged in either procedural knowledge or willingness to collaborate, indicating that the iVR training supported learners equally across professional backgrounds. This study highlights the potential of iVR as a scalable, theory-based approach to IPE that can support interprofessional learning and provide a structured environment for collaborative skill development. Future research should examine sustained effects and comparative effectiveness when iVR is implemented in routine curricular IPE settings beyond controlled study conditions.

## Introduction

### Background

Complex care situations involving emergencies or chronic illnesses with functional impairments present a significant challenge to health care systems. Chronic wounds, for example, are a growing global health problem for countries with an aging population that presents challenges not only for affected patients but for the health care system [1-3]. Providing high-quality wound care requires early and well-coordinated collaboration between clinicians from different professions, such as medicine, nursing, and pharmacy [4]. This is referred to as interprofessional collaboration practice [5].

To prepare future health care professionals for these demands, interprofessional education (IPE) aims to foster shared understanding, role clarity, and joint decision-making among learners from different disciplines [5,6]. Despite its importance, IPE remains challenging to implement in practice. Differences in curricula, institutional separation, and mismatched schedules or experience levels frequently stand in the way of meaningful interprofessional learning opportunities. As a result, the development of interprofessional competencies is frequently inconsistent across curricula [7,8].

Thus, there is a need for innovative interprofessional training opportunities. Immersive virtual reality (iVR) could be a promising approach to enable such an innovative interprofessional training opportunity [9-12]. First, iVR provides a standardized, scalable learning environment [13]. This ensures comparability and consistency across training contexts. By simulating realistic health care scenarios and team interactions [14], iVR eliminates the variability of human instructors, such as fatigue or bias, from the training process. iVR enables repeated practice in a safe environment, protecting patient safety and reducing reliance on the limited availability of real patients [10]. Second, in the context of IPE, iVR eliminates the need for physical co-location of different professional groups [13]. It allows multiple professional perspectives to be integrated within a shared virtual space, creating innovative opportunities for interprofessional learning [15].

Immersion refers to the user’s sense of being present in that environment [16]. The sensory richness and complexity of iVR can impose a cognitive load. Cognitive load is defined as the mental effort required to process and act on information [17]. Thoughtful instructional design can help manage these demands and support learning.

Prior studies suggest that iVR-based training can enhance procedural knowledge [18], foster interprofessional understanding (eg, by strengthening awareness of professional roles and perspectives), and increase learners’ willingness to collaborate [10,19]. However, current IPE approaches still rarely integrate immersive technologies in a structured and theory-based way. Recent reviews call for more rigorous evaluations of digital IPE formats using validated outcomes and theoretically grounded instructional frameworks [20].

### Study Aim: Interprofessional iVR Training

To address this gap, this study investigates the effects of a newly developed interprofessional iVR training focusing on the topic of wound care. Chronic wound care offers a suitable context as it is not yet sufficiently anchored in the medical curriculum [21]. The training engages students from medicine, nursing, and pharmacy, each bringing distinct but complementary knowledge to the collaborative clinical scenario. The theory-based instructional design [22,23] helps learners complete a complex procedure while coordinating effectively across professional roles. We investigate whether this iVR training improves learners’ procedural knowledge (RQ1) and willingness to collaborate (RQ2), and how these outcomes are influenced by learners’ cognitive load experience.

### Cognitive Load and Instructional Design in iVR

Learning outcomes are shaped by the amount and type of cognitive load learners experience, that is, the amount of mental effort required to process information and perform a task according to cognitive load theory [17]. A key distinction is made between extraneous cognitive load (ECL), which results from suboptimal or distracting details and negatively affects learning outcomes. For example, instructions for the wound care process may be displayed in varying positions within the virtual room. Intrinsic cognitive load (ICL) depends on the complexity of the learning task relative to the learner’s prior knowledge and is most beneficial when it reaches a moderate level of challenge. If a task is too simple, learners may disengage, and if it is too difficult, they may become overwhelmed. This suggests an inverted U-shaped relationship between ICL and performance (Seufert [24] explains the effect between ICL and self-regulation). Consequently, cognitive load is an important factor to consider when investigating learning outcomes, particularly in iVR. ECL in iVR can be reduced by adapting the instructional design using computer-based scaffolding [25]. Scaffolding provides temporary support to help learners accomplish tasks beyond their current abilities [25], for example, by breaking down authentic tasks into smaller, clearly identifiable steps, such as a step-by-step guide to wound care, and providing structured instructions. In addition to managing CL, instructional design can also support and structure collaboration through collaboration scripts [22,23]. Such scripts guide specific collaborative activities, for example, discussing wound dressing, and prompt learners to build on each other’s contributions to deepen elaboration. An instructional design for iVR that combines computer-based scaffolding in the form of a step-by-step guide breaking down the wound care process, alongside collaboration scripts, appears particularly promising for improving learning for adequate interprofessional wound care.

### Research Questions and Hypotheses

With respect to RQ1, we hypothesize that students from medicine, nursing, and pharmacy will gain procedural knowledge from the training (H1.1), but that this gain will be negatively moderated by ECL (H1.2) and negatively mediated by ICL, following an inverse U-shaped pattern (H1.3).

In addition, learners may differ in how they benefit from the training due to the different health professions they come from [26]. Prior work suggests that students in medicine, nursing, and pharmacy vary in their clinical experience, communication practices, and expectations regarding interprofessional collaboration [26-28]. These differences may influence how learners engage with the iVR scenario and what they learn from it. Thus, we explore whether the expected knowledge gains differ by learners’ profession (H1.4; exploratory).

With respect to RQ2, we expect students from medicine, nursing, and pharmacy to report increased willingness to collaborate after the training (H2.1), with this increase being negatively moderated by ECL (H2.2). Since learners’ professional backgrounds also shape their collaborative role expectations and prior exposure to teamwork, we explore whether the training affects collaboration willingness differently across professions (H2.3; exploratory). H1.1 to H1.3 and H2.1 to H2.2 were preregistered prior to data collection and are available via Open Science Framework (Multimedia Appendix 1). Findings from this study may inform the development of scalable, theory-based instructional strategies for integrating iVR into IPE.

## Methods

### Sample and Design

The study uses data from an iVR-based training based on a within-subjects design with a pre- and posttest comparison. An a priori power analysis for the preregistered hypotheses, assuming a medium effect size (*f*) of 0.15, with α=.05, and 1-β=.80, yielded a minimum required sample size of n=103. Accordingly, a total of 122 students participated, of whom 6 were excluded due to technical issues with the iVR headsets at the outset, resulting in a final sample of n=116 (N_female_=96, N_diverse_=1; mean age 24.8, SD 4.9 years) of whom 63 were medical students, 31 nursing students, and 22 pharmacy students. Intermediate medical students were included from their third year onwards (mean 4.7, SD 1.1; clinical phase) of a 6-year study program. Nursing and pharmacy students were included from their first study year onwards of a 3.5-year study program (mean 2.1, SD 0.5), respectively, of a 4-year study program (mean 2.1, SD 0.8 years). Participants were scheduled to train in interprofessional triads. In total, training was organized into 30 planned triads; due to anticipated but absent participants and the need to merge remaining attendees to keep sessions feasible, the realized training groups comprised 9 dyads and 2 tetrads. Previous experience with wound care was reported by 23 medical students, 4 nursing students, and 3 pharmacy students. Previous experience with VR was reported by 16 medical students, 4 nursing students, and 3 pharmacy students.

### iVR Environment/learners’ Task

The authors with backgrounds in medicine, psychology, learning sciences, and nursing education developed an iVR training in collaboration with medverse GmbH. The contents and procedures had been developed for the iVR environment based on established clinical standards [29] and were agreed upon through a consensus among wound care experts from medicine, nursing, and pharmacy, incorporating profession-specific perspectives. The iVR is divided into 3 sections: preparation, patient contact, and follow-up.

In the first section, preparation, participants find themselves in front of the patient’s room. There, the senior physician presents the case using the SBAR (Situation-Background-Assessment-Recommendation) framework, which is a well-established standard for structured patient handovers [30]. SBAR improves nurse–physician communication and reduces unexpected death. The patient is an 81-year-old man with a category 3 pressure ulcer (EPUAP classification [31]) on his right heel. The ulcer developed following surgical treatment of a femoral neck fracture with total hip arthroplasty, after which the patient experienced severe postoperative pain and markedly limited mobility, leading to prolonged pressure exposure. At the time of the scenario, the wound measured 5.5 cm × 3 cm, was painful, moderately exudative, and showed scattered fibrin deposits. The clinical situation required coordinated wound care, appropriate pain management, pressure relief, and mobilization planning. Multimedia Appendix 2 provides a detailed description of the case materials used. The participants then gather for an initial team meeting to discuss the latest wound documentation. This is presented in written form and includes a detailed description of the wound (eg, size), previous treatment (eg, dressing), and the patient’s reported pain level.

In the second section, patient contact, participants enter the patient’s room, introduce themselves, explain their purpose, and ask about the patient’s pain level. After, learners are guided to discuss pain management, review current therapy, and possible therapy adjustments. Further, they consider potential changes to the patient’s position, as well as mobilization strategies. An animation demonstrates bandage removal. Participants examine the removed dressing and its documentation (eg, moisture), inspect the wound, and select a cleaning method and product from 2 options. They then receive feedback on whether their choice was correct. An animation illustrates the cleaning process. They photograph the wound, review it after cleaning with the written documentation, and decide on wound edge protection and dressings, again from 2 options, with feedback. Another animation shows the application of the bandage. Finally, they discuss patient instruction, considering the information needed for wound self-care, including infection risks and preventive measures.

**Figure 1.**
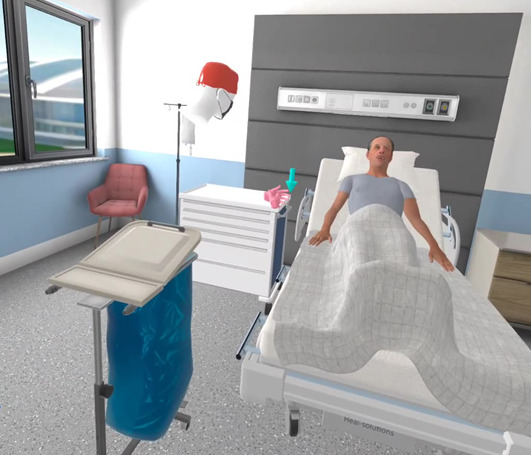
Participant interacting with the patient in the virtual patient room.

In the final section, follow-up, participants leave the patient’s room and gather for a final team meeting. They discuss the treatment provided and any further care required, such as intervals for changing the dressing, and the involvement of other professional groups. The iVR training concludes at this point. Figures1  2 display screenshots from the iVR environment at different time points. The training covers adherence to German hygiene standards [32] in the wound care process. Participants learn when to disinfect their hands and surfaces, when to put on and change protective clothing, including gloves, and how to position equipment correctly.

**Figure 2.**
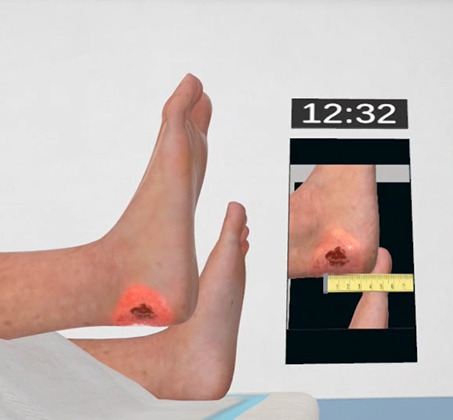
Detailed view of the pressure ulcer on the patient’s right heel.

### Procedure

The study was conducted as part of a larger data collection in June 2025. Recruitment took place via semester kick-off events, lectures, student learning platforms, messaging groups, and campus notices. Learners registered individually and were assigned to interprofessional triads (at least 2 different professions per group). Participation was voluntary; written informed consent was obtained prior to participation. Each participant received €20 (US $23.22) via bank transfer after participation, and compensation was fixed and not performance-based. Each session lasted approximately 2 hours and comprised a pretest, a learning phase (iVR training unit), and a posttest. Participants began individually with a tablet-based pretest covering demographics, prior experience with iVR and wound care, procedural knowledge, and willingness to collaborate. After standardized equipment instructions, triad members sat in the same room in a semicircle, put on their iVR headsets, took their controllers, and completed the iVR training unit (average duration: 25 min), communicating verbally but without facial cues. This was followed by a posttest assessing cybersickness, procedural knowledge, cognitive load, and willingness to collaborate. In addition, a second iVR training unit (average duration: 15 min) was administered, followed by a second posttest (posttest 2). The iVR training was developed based on shared learning objectives targeting both procedural wound care and interprofessional collaboration. The instructional design comprised 2 consecutive levels with constant procedural content but reduced collaborative guidance in the second level. Procedural knowledge was assessed at pretest and posttest 1, as the stepwise wound-care sequence remained unchanged across levels and the sequencing task was susceptible to retest effects. Willingness to collaborate was assessed at all 3 time points to allow examination of potential changes across repeated interprofessional engagement and varying degrees of collaborative support. Analyses involving posttest 2 are reported as exploratory.

Missing data resulted from technical issues (eg, temporary internet connectivity problems leading to headset malfunctions and occasional questionnaire interruptions) and time constraints, particularly affecting posttest 2. No systematic dropout related to study variables was observed.

### Instructional Support

**Figure 3.**
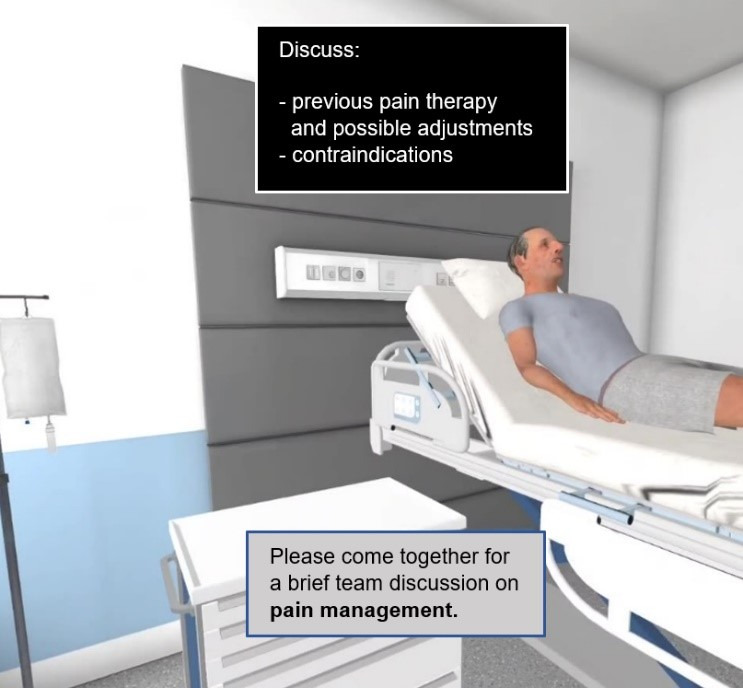
Example of a meta-prompt with a checklist in the immersive virtual reality (iVR) to guide participants’ collaboration.

Throughout the iVR training, participants were supported with scaffolding and collaboration scripts implemented as prompts. Scaffolding was provided via step-by-step tooltips aligned with the wound care process (eg, “Adjust the bed to working height”), displayed as pop-ups at hand level. Collaboration scripts were implemented at 7 predefined points identified with external wound experts from medicine, nursing, and pharmacy. For example, participants received the meta-prompt “Please come together for a brief team discussion on pain management.” Discussions were structured by a checklist (eg, “Discuss previous pain therapy and possible adjustments”), both displayed above the patient’s bed in the iVR environment (Figure 3).

In the second iVR training unit, only the meta-prompt was provided to encourage more self-structured discussion. All prompts and checklists are listed in Multimedia Appendix 3. Progression required completing all actions; some steps were triggered automatically (eg, tapping the bed), while checklist items required active confirmation via a “Next” button.

### Measures

Procedural knowledge was assessed based on the expert-based wound care steps in the pre- and post-tests using a drag-and-drop sequencing task (Multimedia Appendix 4). The test covered all 12 steps of the wound care process. Each step was described in concise, clear wording (eg, “Assess the wound and change gloves,” “Select wound edge protection and dressing, disinfect hands, put on gloves, dress the wound”). Responses were scored using Kendall τ, a rank correlation coefficient that quantifies the correspondence between the participant’s ordering and the correct reference ordering. Higher values indicate better procedural knowledge, ranging from −1 (perfectly reversed order) to +1 (perfectly correct order), with values around 0 reflecting largely unsystematic or random ordering. Thus, positive shifts in Kendall τ reflect increasingly correct sequencing of clinically relevant procedural steps. Because the task required ordering the complete procedure as a whole, individual steps were not scored independently, and step-level error rates were not separately coded.

Willingness to collaborate was measured using a current German translation (validation study ongoing) of the Interprofessional Socialization and Valuing Scale (ISVS) [33]. Willingness to collaborate is considered a key construct in interprofessional contexts, where it is critical for team effectiveness. This ability develops through a process of interprofessional socialization [33], during which clinicians adopt professional and interprofessional identities, recognize the value of other disciplines’ contributions, and function confidently as collaborative team members. This socialization lays the groundwork for the delivery of high-quality, integrated care in complex clinical settings. Willingness to collaborate was assessed at 3 measurement timepoints (pretest, posttest 1, and posttest 2). Accordingly, 3 corresponding versions of the ISVS were used: ISVS-9A (9 items) at pretest, ISVS-9B (9 items) at posttest 1, and ISVS-21 (21 items) at posttest 2. These are validated equivalent forms of the same underlying construct. The two 9-item versions were specifically developed for repeated-measures designs and have demonstrated strong agreement and measurement invariance with each other. The use of different but equivalent forms was a deliberate methodological choice to reduce response burden and potential repetition effects across repeated measurements while maintaining comparability across timepoints. An example item from set A was “I am able to share and exchange ideas in a team discussion” and “I feel comfortable in describing my professional role to another team member.” All items were rated on a 7-point Likert scale (1=does not apply at all, 7=fully applies; 0=no answer). Negatively worded items were reverse-coded so that higher scores indicate greater willingness to collaborate. For each participant, we calculated the mean score across all nonmissing items. Internal consistency was good (*ω*_pre_=0.77; *ω*_post1_=0.77).

Cognitive load was assessed using the scale by Klepsch et al [34]. Participants rated ICL and ECL on a 7-point Likert scale ranging from absolutely wrong to absolutely right. ICL was measured with 2 items (eg, “For this task, many things needed to be kept in mind simultaneously”; “This task was very complex”), with *ω*=0.63. ECL was measured with 3 items (eg, “The instructions and explanations during this task were very unclear”; “During this task, it was difficult to recognize and link the crucial information”), with *ω*_post1_=0.67. For each participant, we calculated the mean score across all nonmissing items.

Cybersickness was assessed as a control variable using the German version of the Virtual Reality Symptom Questionnaire (VRSQ-G) [35]. The questionnaire contains 9 symptoms (eg, fatigue), each rated on a 4-point scale (0=not at all to 3=very much). Internal consistencies were good (*ω*_post1_=0.87).

### Analyses

To address RQ1 and RQ2, we examined changes in the primary outcomes across measurement points and then tested whether these changes were associated with cognitive load while controlling for cybersickness. For RQ1, we examined whether procedural knowledge increased from pretest to posttest 1 (H1.1) and whether this change was moderated by ECL (H1.2). In addition, we tested the proposed nonlinear association between ICL and procedural knowledge (H1.3). Profession-related differences were explored (H1.4).

For RQ2, willingness to collaborate was assessed at pretest, posttest 1, and posttest 2. We examined whether willingness to collaborate increased over time (H2.1) and tested whether these changes were moderated by ECL while controlling for cybersickness (H2.2). Analyses including posttest 2 were conducted exploratorily, as this third measurement point was not part of the preregistered hypotheses. Profession-related differences were explored (H2.3).

Change and moderation hypotheses were tested using repeated-measures analyses of covariance (ANCOVAs). For RQ1 (procedural knowledge), analyses included 2 measurement points (pretest and posttest 1, corresponding to the first iVR training level). For RQ2 (willingness to collaborate), repeated-measures ANCOVAs included 3 measurement points (pretest, posttest 1, and posttest 2). For moderation analyses, ECL was included as a covariate and cybersickness as a control variable. The proposed nonlinear effect of ICL was examined using a mediation model including a quadratic term (ICL²). Exploratory profession-related differences were examined using ANCOVAs.

All analyses were based on the preregistered analysis plan described above. While the theoretical assumptions and target constructs remained unchanged, minor refinements were made to align the analytic implementation with the final study design and improve interpretability.

Because participants were trained in interprofessional triads, potential clustering effects were examined using linear mixed models with random intercepts for participants and triads. The necessity of multilevel modeling was evaluated using the design effect [36]. When the design effect was smaller than two, single-level models were retained as primary analyses, and mixed models were estimated as robustness checks.

Missing data were handled according to the estimation method used in each analysis. Repeated-measures ANCOVAs were estimated using listwise deletion within each model. Linear mixed models were estimated using restricted maximum likelihood and included all available observations. Mediation analyses were conducted using complete cases as implemented in Jamovi. All analyses were conducted in Jamovi (version 2.0; The Jamovi project, 2024) and can be retrieved from the Open Science Framework (Multimedia Appendix 1).

### Ethical Considerations

The Ethics Committee of the Medical Faculty at LMU Munich confirmed that no formal ethics review was required for this study (number 25-0754-KB). The study was conducted in accordance with the ethical principles of the Declaration of Helsinki (WMA). All participants received information about the study procedures, the voluntary nature of participation, and their right to withdraw from the study at any time without disadvantage. Informed consent was obtained from all participants prior to participation. Privacy and confidentiality were maintained throughout the study. Data were collected and analyzed in pseudonymized form, and no directly identifying information is reported in this manuscript. Each participant received €20 (US $23.22) via bank transfer after participation. Compensation was fixed and not performance-based.

## Results

### Overview

Professions were distributed across the sample. Table 1 contains descriptives of the study variables with the respective correlations in Table 2.

**Table 1.** Descriptive statistics of the study variables.

Variable	N	Missing	Mean	SD	Min	Max	*P* (Shapiro-Wilk)
Procedural knowledge pre	116	0	−0.111	0.0653	−0.333	0.0606	<.001
Procedural knowledge post	110	6	0.0501	0.0571	−0.212	0.242	<.001
Willingness to collaborate pre	100	16	5.6	0.631	3.67	6.78	.23
Willingness to collaborate post	97	19	5.58	0.666	4.11	7.0	.38
Willingness to collaborate post 2	90	26	5.74	0.608	3.78	6.89	.055
ECL^a^ post	110	6	2.53	1.09	1.0	5.67	<.001
ICL^b^ post	110	6	3.26	1.2	1.0	6.0	<.001
Cybersickness post	110	6	1.84	0.66	1.0	4.0	<.001

a ECL: extraneous cognitive load.

b ICL: intrinsic cognitive load.

**Table 2.** Correlations between the study variables. Pearson correlations are reported.

Variable	1	2	3	4	5	6	7	8
Procedural knowledge pre								
Procedural knowledge post	−.045							
Willingness to collaborate pre	−.161	.020						
Willingness to collaborate post	.006	.104	.527^a^					
Willingness to collaborate post 2	−.133	−.108	.567^a^	.632^a^				
ICL post	.000	−.063	.158	.165	.181			
ECL post	.029	.080	−.180	−.272^b^	−.180	.113		
Cybersickness post	−.018	.126	−.213^c^	−.202^c^	−.040	−.017	.343^a^	

a *P*<.001.

b *P*<.01.

c *P*<.05.

Because participants were trained in interprofessional triads, we examined whether clustering at the triad level affected the estimated effects. Linear mixed models including random intercepts for participants and triads were estimated for both outcomes. For procedural knowledge, the intraclass correlation at the triad level was small (intraclass correlation coefficient [ICC]=0.035). For willingness to collaborate, the triad-level variance component was estimated at zero (ICC=0.000). The resulting design effects were below the commonly discussed threshold of two. As the pattern of fixed effects remained unchanged in the mixed models, the preregistered ANCOVAs are reported as the primary analyses.

### RQ1: Effects of iVR Training on Procedural Knowledge and the Role of Cognitive Load

To address RQ1, we first examined whether procedural knowledge changed from pretest to posttest 1. Mean values increased from −0.11 at pretest to 0.05 at posttest, indicating that the ordering of steps changed from a systematic error to a level slightly above random. This change was tested using a repeated-measures ANCOVA (n=110) with measurement point (pretest, posttest) as a within-subjects factor, ECL as a covariate, and cybersickness as a control variable. The analysis revealed a significant main effect of measurement point, *F*_1, 107_=26.19, *P*<.001, η²=.08, indicating a gain in procedural knowledge. Thus, H1.1 is supported. On average, students moved from a slightly systematically incorrect ordering at pretest (negative Kendall τ) to a predominantly correct ordering at posttest (positive Kendall τ), reflecting a meaningful reorganization of the clinically relevant wound-care sequence.

A post-hoc comparison confirmed that procedural knowledge was significantly higher at posttest compared with pretest (*t*_107_=19.60, *P*<.001), with a mean difference of 0.163. Neither the interaction between measurement point and ECL (*F*_1, 107_=.00, *P*=.99, η²=.00) nor the interaction between measurement point and cybersickness (*F*_1, 107_=0.89, *P*=.35, η²=.00) was significant. Thus, H1.2 was not supported. H1.3 posited a quadratic mediating effect of ICL on procedural knowledge. The mediation model (n=110), including ICL^2^ showed no statistically significant effects. There was no indirect effect (*b*=0.00, SE=0.00, *Z*=0.014, *P*=.99), no direct effect (*b*=–0.04, SE=0.09, *Z*=–0.472, *P*=.64), and no total effect (*b*=–0.04, SE=0.09, *Z*=–0.470, *P*=.64). Thus, H1.3 was not supported. Further, the exploratory analysis (n=104) revealed no significant differences between professions (*F*_2, 101_=0.07, *P*=.93, η²=.00). Thus, H1.4 was not supported.

### RQ2: Effects of iVR Training on the Willingness to Collaborate and the Role of Cognitive Load

To address RQ2, we examined whether willingness to collaborate changed from pre to posttest. Mean willingness to collaborate was 5.6 at pre and 5.58 at posttest. A repeated measures ANCOVA (n=83) with measurement point (pre, posttest) as a within-subjects factor, ECL as a covariate, and cybersickness as a control variable revealed no significant main effect of measurement point (*F*_1, 80_=3.55, *P*=.06, *η²*=.01), indicating no measurable change in willingness to collaborate from pre- to posttest and thus not supporting H2.1. A post hoc comparison confirmed that willingness to collaborate did not differ significantly between pre- and posttest (*t*_80_=–0.54, *P*=.59), with a mean difference of –0.036. Neither the interaction between measurement point and ECL was significant (*F*_1, 80_=3.71, *P*=.058, *η²*=.01), nor the interaction between measurement point and cybersickness (*F*_1, 80_=0.51, *P*=.477, *η²*=.001). Thus, H2.2 was not supported. Exploratory analyses (n=83) including the third measurement point (posttest 2) showed that willingness to collaborate was significantly higher at posttest 2 than at posttest 1 (*t*_64_=3.16, *P*=.007, mean difference=0.202) and significantly higher at posttest 2 than at pretest (*t*_64_=2.47, *P*=.043, mean difference=0.167). No significant difference emerged between pretest and posttest 1 (*t*_64_=–0.48, *P*=.882, mean difference=–0.034). Further, no significant differences were found between professions (*F*_2, 75_=0.23, *P*=.794, *η²*=.00). Thus, H2.3 was not supported.

## Discussion

### Principal Findings

This study investigated the effects of a newly developed iVR training on wound care, completed in interprofessional groups of students from medicine, nursing, and pharmacy. Specifically, we examined whether the training improves learners’ procedural knowledge and willingness to collaborate, and how these outcomes are shaped by cognitive load.

With respect to RQ1, learners demonstrated a large and statistically significant gain in procedural knowledge from the pre to posttest, thus confirming H1.1. Importantly, this shift reflects a move from slightly systematically incorrect sequencing at baseline to predominantly correct procedural ordering after the training.

This finding is consistent with previous studies showing that iVR can promote procedural knowledge [18] by simulating realistic clinical processes and enabling repeated, safe practice [10]. The effect was observed across medical, nursing, and pharmacy students who all started with low levels of prior knowledge and showed marked gains after the training, independent of their professional background. Contrary to H1.2, ECL did not moderate this knowledge gain. This may have been because ECL was managed effectively through the scaffolding [25] and collaboration scripts [22,23] implemented in the iVR. More precisely, we implemented scaffolding by providing a step-by-step structure that broke wound care down into smaller, more manageable steps. This guided learners through the entire process, ensuring that they did not overlook essential actions, since they had to confirm or interact in order to proceed. In addition, animations of complex handling tasks (eg, unwrapping a bandage) reduced task complexity, helping learners avoid overload and focus on the essential learning content. Similarly, some hygiene steps (eg, hand disinfection) required only confirmation rather than haptic execution at a dispenser, further lowering unnecessary demands. Our structured design may have enabled learners with limited prior knowledge to concentrate on the essential steps, possibly without becoming cognitively overloaded. In line with cognitive load theory [17], such instructional support may contribute to a reduction in ECL, enabling learners to focus on the task itself. Nevertheless, caution is required when interpreting this. As outlined in the Limitations section, modest subscale reliability and self-report constraints may have limited sensitivity. Thus, the absence of cognitive load effects should not be attributed solely to instructional load control through the instructional design.

According to H1.3, ICL was expected to show an inverted U-shaped effect, but this was not supported. One possible explanation is that all learners experienced a moderate level of ICL, enabling them to benefit from the learning environment and achieve overall learning gains. This interpretation is consistent with previous theoretical models’ [37] findings on the relationship between ICL and self-regulation. In line with this pattern, no profession-related differences were found in terms of gains in procedural knowledge either. Contrary to H1.4, this indicates that the iVR training effectively fostered procedural knowledge across different professional backgrounds, providing comparable benefits for medical, nursing, and pharmacy students.

With respect to RQ2, there was no significant increase in willingness to collaborate between the pre- and posttest, which provides no support for H2.1. However, the exploratory analysis revealed an improvement in learners’ willingness to collaborate after the second iVR session. This suggests that a single 25-minute iVR session may be insufficient to change learners’ attitudes towards collaboration, as these attitudes are developed over time through an interprofessional socialization process [33,38]. An attitudinal change may require more time to unfold. This pattern also aligns with the idea that interprofessional values, role understandings, communication skills, and teamwork competencies develop gradually over an extended period of training and interaction within interprofessional teams [6,33]. A single exposure, even within an immersive and structured environment, may not be sufficient to induce measurable change on a self-report scale. To increase sensitivity for this outcome, future studies could incorporate repeated or longitudinal interprofessional VR sessions, structured debriefings, or explicit perspective-taking tasks to deepen reflection on professional roles. In addition, combining self-report measures with behavioral indicators of collaboration quality may provide a more fine-grained assessment of interprofessional development.

Contrary to H2.2, ECL did not moderate changes in willingness to collaborate. One possible explanation might be that learners were able to effectively manage the task with the help of instructional support [22,23]. In our study, we implemented collaboration scripts by structuring the team process, indicating when discussions should take place, and providing checklists detailing the issues to be addressed in each meeting. Learners therefore received individual prompts such as “Please come together for a brief team discussion on pain management,” and structured checklists highlighted the topics that they needed to discuss. Learners could progress to the next step only after confirming that they had addressed all points. However, again, caution is required when interpreting the absence of ECL-related effects on willingness to collaborate as evidence of effective instructional load control. Furthermore, contrary to H2.3, no differences in willingness to collaborate based on profession were observed. This indicates that nursing, pharmacy, and medical students increased their willingness to collaborate similarly after the iVR training. Overall, iVR training led to an immediate and substantial increase in procedural knowledge, whereas willingness to collaborate improved only after repeated exposure, indicating that attitudinal outcomes require a longer time frame to develop. While the instructional design may have contributed to structuring task demands, this finding should be interpreted in light of the modest reliability of the cognitive load subscales and the limitations of self-report measures in immersive and collaborative VR environments.

### Limitations and Further Research

Several limitations should be considered when interpreting the findings. The study used a single-group pretest–posttest design without a comparison condition. This design was chosen to evaluate the feasibility and initial learning effects of a newly developed interprofessional VR training under controlled conditions. While causal attribution to the instructional format itself cannot be established within this design, and alternative explanations such as testing or maturation effects cannot be fully excluded, it allows a focused examination of within-group development during early-stage implementation. Comparative and noninferiority VR trials exist in other domains [39]; however, such designs are not always required at early stages of instructional development, particularly when the primary aim is to examine feasibility, acceptability, and initial learning processes within a new interprofessional context. This study, therefore, addressed a distinct research question focused on interprofessional implementation and within-group development. Future research should build on these findings using controlled designs to determine under which conditions iVR provides added value within IPE.

The collaborative interaction relied exclusively on verbal communication without nonverbal cues, which may limit ecological validity compared with in-person interprofessional teamwork. Future studies should examine how integrating nonverbal communication elements, for example, through augmented or mixed-reality environments, influences interprofessional collaboration processes.

Moreover, this study focused on learning-related outcomes and did not systematically evaluate structural or economic aspects of implementation. At the same time, a key structural advantage of iVR is that, once developed and technically implemented, it can be deployed independently of dedicated simulation centers, potentially lowering access barriers for institutions with limited simulation infrastructure. Future research should therefore examine how such VR-based formats can be sustainably integrated into IPE and evaluate their scalability and comparative effectiveness under routine conditions.

Measurement-related constraints should also be acknowledged. Internal consistency for cognitive load was moderate, and self-report measures may only partially capture the multidimensional demands of collaborative iVR environments, which combine sensory immersion, interface navigation, clinical decision-making, and real-time interprofessional coordination. In such settings, learners may experience overlapping cognitive, social, and perceptual demands that are difficult to fully disentangle through brief retrospective ratings. Willingness to collaborate was assessed using validated equivalent ISVS forms across time points, which reduces repetition effects and participant burden but limits strict item-level longitudinal comparability, particularly for the exploratory third measurement.

In addition, procedural knowledge was assessed at pretest and posttest 1 to avoid retest effects of the sequencing task and to align with the preregistered focus on the first training level. This design does not permit conclusions about retention or additional gains after repeated exposure. The study also did not include process-based indicators of collaboration quality, as the primary focus was on attitudinal and knowledge-related outcomes. Future research could complement these measures with longitudinal assessments, parallel or alternative knowledge indicators across multiple training iterations, and process-oriented metrics of interprofessional interaction.

Further, the sample consisted of students whose professional identities are still developing. While this limits direct transferability to experienced clinicians, early professional socialization is a core objective of IPE. This suggests that iVR training should be delivered as a series of sessions rather than as a one-off event, as repeated exposure may help learners build trust and appreciation for other professions and gradually increase openness and readiness to collaborate. Future research should therefore examine how collaborative attitudes and skills develop longitudinally across repeated IPE experiences and at more advanced stages of professional training. While our training already includes an in-built debriefing component, this could be complemented by facilitated interprofessional reflection outside the VR environment. In addition, collaboration quality could be captured via observable and codable behavioral indicators (eg, closed-loop communication) instead of being assessed solely through self-report measures.

Finally, the training focused on a single wound-care case with structured decision pathways to maintain manageable cognitive demands within the immersive environment. This design enabled a focused examination of interprofessional coordination in a clearly defined clinical scenario. At the same time, clinical wound management is inherently context-dependent, shaped by patient-specific factors, institutional routines, and practical constraints, and different constellations may warrant alternative decisions.

Future work should therefore extend immersive interprofessional simulations to diverse cases and decision pathways in order to examine transfer across clinical contexts.

### Implications

The findings of this study have several implications for the design and implementation of IPE. First, the substantial gain in procedural knowledge among all learners indicates that iVR training can be used effectively. Integrating such training early on into the curriculum could help to ensure that students of medicine, nursing, and pharmacy acquire a shared foundation precisely in domains where their prior knowledge is typically low but clinical relevance is high, such as wound care.

Second, the results suggest that fostering interprofessional attitudes needs repeated, well-structured learning opportunities. In our study, willingness to collaborate only increased after the second training session, demonstrating that such changes do not occur immediately. This suggests that iVR training should be delivered as a series of sessions rather than as a one-off event. Repeated exposure allows learners to build trust and appreciation for other professions and fosters a gradual shift towards openness and readiness to collaborate. Third, the instructional design features used, such as step-by-step scaffolding throughout the case and structured collaboration prompts via meta-prompt plus checklist, proved to be feasible and effective. These features can serve as instructional blueprints for other interprofessional learning contexts. These elements reduce unnecessary demands and provide clear guidance for interaction, enabling learners to focus on essential steps and collaborative decision-making. These elements can be flexibly applied to iVR scenarios, which is more difficult to achieve in real-world settings with simulated patients.

Finally, the study highlights the curricular and organizational potential of iVR. Beyond acute scenarios, iVR enables systematic training in areas of practice that are otherwise difficult to integrate into curricula and where learners typically have little prior exposure. Its standardization and relatively low resource requirements make it a scalable approach to IPE that can be implemented even in institutions without large simulation centers, thereby broadening access to authentic interprofessional learning opportunities.

### Conclusions

This study provides initial evidence that iVR can support IPE by improving procedural knowledge in a standardized, theory-based learning environment. Students from medicine, nursing, and pharmacy showed significant gains in procedural knowledge after the iVR wound care training, suggesting that immersive, scaffolded, and script-based instructional designs may facilitate complex skill acquisition across professional groups. In contrast, willingness to collaborate increased only after repeated exposure, suggesting that attitudinal and interprofessional competencies require sustained, iterative learning experiences rather than single training sessions. These training effects were independent of the learners’ professions. Overall, the findings highlight the potential of iVR as a scalable approach for integrating interprofessional training into health care curricula. Repeated and structured iVR sessions could complement existing IPE programs, particularly in domains where task coordination and shared decision-making are central to patient care. Future research should examine long-term iVR effects on collaboration attitudes and behavior, extend iVR evaluations to various practicing professionals, and develop refined measurement tools for cognitive and collaborative demands in iVR for IPE.

## Supplementary material

10.2196/85139 Multimedia Appendix 1 Preregistration and analyses.

10.2196/85139 Multimedia Appendix 2 Patient presentation and wound documentation.

10.2196/85139 Multimedia Appendix 3 Instructional support.

10.2196/85139 Multimedia Appendix 4 Procedural knowledge test.

## Abbreviations

ANCOVA: analysis of covariance
ECL: extraneous cognitive load
ICC: intraclass correlation coefficient
ICL: intrinsic cognitive load
IPE: interprofessional education
ISVS: Interprofessional Socialization and Valuing Scale
iVR: immersive virtual reality
SBAR: Situation-Background-Assessment-Recommendation
VRSQ-G: Virtual Reality Symptom Questionnaire - German version
