# Supplementary material for: Interprofessional Training in Virtual Reality for Health Care: Experimental Study on Procedural Knowledge and Willingness to Collaborate
Source: JMIR Med Educ. 2026 May 27;12:e85139. doi: 10.2196/85139 (PMC13215666; doi:10.2196/85139)
Supplement: Multimedia Appendix 1 [file mededu-v12-e85139-s001.pdf]

Preregistration: https://osf.io/dwxus/?view_only=79c291300a1f412fb4c86df4a5efaaf4

Analyses:
https://osf.io/3cyxn/overview?view_only=825d11f4a149427583f180b95176ca55
