# Supplementary material for: Interprofessional Training in Virtual Reality for Health Care: Experimental Study on Procedural Knowledge and Willingness to Collaborate
Source: JMIR Med Educ. 2026 May 27;12:e85139. doi: 10.2196/85139 (PMC13215666; doi:10.2196/85139)
Supplement: Multimedia Appendix 2 [file mededu-v12-e85139-s002.pdf]

Multimedia Appendix 2. Patient presentation and wound documentation.

This appendix contains the patient presentation delivered by the senior physician, as well as the wound documentation displayed on the board next to the patient's door:

Hello, my name is Dr. Maier. I am the senior physician. I would like to introduce you to our patient, Mr. Peter Schneider. He is in his early 80s and is currently hospitalized with a pressure ulcer on his right heel. Ten days ago, he suffered a femoral neck fracture, which was treated with surgery involving a total endoprosthesis. Unfortunately, he could only be mobilized to a very limited extent after the operation due to severe pain, which led to the development of the pressure ulcer. We are not aware of any preexisting conditions or allergies. His current laboratory values are normal. The pressure ulcer is now at an advanced stage and highly sensitive to pain. Mr. Schneider received one gram of Novalgin thirty minutes ago. I recommend thoroughly cleansing the wound and applying a dressing that absorbs exudate well. Appropriate pain management and close monitoring of the wound are also important. Additionally, a mobilization plan should be developed.

Wound documentation (three days ago):

Name: Peter Schneider, Date of birth: January 21, 1944

Wound type: Pressure Ulcer on Right Heel

Wound classification: Category 3, according to the EPUAP

Wound pain: 6/10

Size: 5.5 cm x 3 cm

Wound environment: dry

Wound edge: smooth

Exudate: Moderate, serous

Wound bed: Scattered fibrin deposits

Wound odor: none

Wound irrigation: 0.9% NaCl

Multimedia Appendix 2. Patient presentation and wound documentation.

Wound dressing: PU foam dressing, Secondary dressing: Gauze bandage

Other therapies: Pressure relief and mobilization three times daily at the edge of the bed
