# Supplementary material for: Interprofessional Training in Virtual Reality for Health Care: Experimental Study on Procedural Knowledge and Willingness to Collaborate
Source: JMIR Med Educ. 2026 May 27;12:e85139. doi: 10.2196/85139 (PMC13215666; doi:10.2196/85139)
Supplement: Multimedia Appendix 3 [file mededu-v12-e85139-s003.pdf]

Multimedia Appendix 3. Instuctional support.

This appendix contains all instructional support in the form of step-by-step tooltips for the wound care process, as well as the collaboration points with meta-prompts (shown in bold) and checklists. *Further essential information* is marked in italics:

Please come together for a brief team discussion on wound documentation.

Discuss the wound documentation. You can see it on the board here.

Disinfect your hands.

Knock on the patient's door and enter the room.

Greet the patient and introduce yourself.

Explain to the patient why you are there.

Ask them to rate their pain on a scale of 1 to 10.

Please come together for a brief team discussion on pain management.

Discuss:

- Previous pain therapy and possible adjustments
- Possible contraindications

Ask the patient if they have any questions.

Look around the treatment room and identify the error *(the window must be closed).*

Prepare in accordance with hygiene standards (disinfect, put on a protective gown, mask, and gloves).

Clean the work surface on the dressing trolley.

Place the dressing trolley and waste bin in a hygienic triangle. Start with the table.

Now place the waste bin.

Change gloves in accordance with hygiene standards (disposal, disinfection, new gloves).

Adjust the bed to working height.

Please come together for a brief team discussion on positioning.

Multimedia Appendix 3. Instuctional support.

Discuss:

• Current positioning and any changes

• Positioning plan and mobilization strategy

Place the absorbent pad under the heel.

Remove the dressing.

The assessment of the dressing material reveals the following: Secondary dressing (gauze bandage): Moistened. Viscous yellowish exudate is visible. Slightly displaced. Primary dressing (PU foam dressing): Moistened. No evidence of bleeding.

Examine the wound individually.

Dispose of your gloves in accordance with hygiene standards (disposal and disinfection).

Please come together for a brief team discussion on wound cleaning method and product.

Discuss and decide on the following:

• Wound cleaning methods: non-touch technique with tweezers vs. sterile gloves

• Suitable wound cleansing products (NaCl 0.9% vs. Octenisept).

Solution: Both cleansing methods are correct. Today, we will use the non-touch technique with tweezers. The correct wound cleansing product is NaCl. Prepare the treatment trolley with tweezers and NaCl solution. [The 'correct/incorrect' feedback is a scenario-specific simplification. In this case, the wound documentation and assessment show no signs of infection, so 0.9% NaCl is considered sufficient. Depending on local clinical guidelines and the clinical context, antiseptic solutions may also be appropriate.]

Put on new gloves according to hygiene standards (disinfect and put on new gloves).

Clean the wound.

Dispose of your gloves according to hygiene standards.

Take a photo of the wound.

Take another look at the wound's condition.

Multimedia Appendix 3. Instuctional support.

The assessment reveals: Wound Environment: Dry and non-irritated with an imprint of an old dressing visible. Wound edge: Partially epithelialized and partially macerated. Exudate: Heavy and viscous. The wound dressing is not sufficiently absorbent.Wound bed: Granulation tissue, incipient epithelialization, partial fibrin coating, and possible biofilm. Odor: Currently not assessable.

Please come together for a brief team discussion on wound edge protection and dressings.

Discuss and decide on:

• Suitable wound edge protection: Cavilon (acrylate-based skin protection film) vs. zinc ointment

• Suitable wound dressing (PU foam dressing vs. sterile compress).

Solution: The correct wound edge protection is Cavilon and the correct dressing is the PU foam dressing. Now, prepare the dressing trolley. [The "correct/incorrect" feedback reflects a scenario-specific simplification; in this case, the wound documentation indicates increased exudate and a partially macerated periwound, and a PU foam dressing with adhesive fixation is planned, therefore a barrier film (Cavilon) is selected to protect the wound edges. Depending on clinical context and local guidelines, alternatives (eg, zinc ointment or a sterile compress) may also be appropriate in other situations.]

Put on new gloves in accordance with hygiene standards (disinfection, new gloves). Dress the wound. Tidy up your work area.

Dispose of your gloves in accordance with hygiene standards (disposal, disinfection).

Lower the patient's bed.

Please come together for a brief team discussion on patient instructions.

Discuss and decide on:

• Important information on wound self-care

• Information on infection risks and preventive measures.

Say goodbye to the patient, then leave the room.

Please come together for a brief team debriefing.

Multimedia Appendix 3. Instuctional support.

Consider and discuss:

- Reflection on the wound care performed
- Further steps, dressing technique, and dressing change interval
- Involvement of other professional groups
