# Supplementary material for: Interprofessional Training in Virtual Reality for Health Care: Experimental Study on Procedural Knowledge and Willingness to Collaborate
Source: JMIR Med Educ. 2026 May 27;12:e85139. doi: 10.2196/85139 (PMC13215666; doi:10.2196/85139)
Supplement: Multimedia Appendix 4 [file mededu-v12-e85139-s004.pdf]

Multimedia Appendix 4. Procedural knowledge test.

This appendix contains the procedural knowledge test with its 12 steps that were previously presented in random order and had to be arranged in the correct sequence:

Imagine that you are treating a patient's wound in a clinical setting.

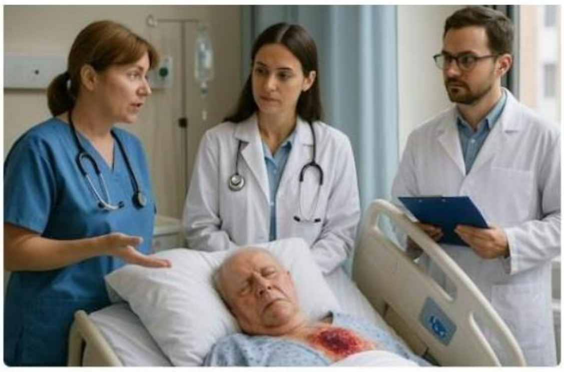

Below are the 12 steps of wound care:

**1** Reposition the patient, place an absorbent underpad, and remove the dressing.
**2** Document the wound by taking a photo and reassess it.
**3** Educate the patient on self-care of the wound, clarify any questions, and take your leave.
**4** Select wound edge protection and dressing, disinfect hands, put on gloves, and dress the wound.
**5** Disinfect work surfaces, set up the hygienic triangle, change gloves, and adjust the bed to working height.
**6** Assess and dispose of the old dressing, assess the wound, and change gloves.
**7** Ask about the current pain level, adjust pain therapy if necessary, disinfect hands, and put on gown, mask, and gloves.
**8** Reflect on the wound care performed, plan next steps, and involve other professional groups if necessary.
**9** Greet the patient, introduce yourself, and state the reason for your visit.
**10** Choose the wound cleansing method and product, perform wound cleansing, dispose of gloves, and disinfect hands.
**11** Present the patient case in front of the patient's room, disinfect hands.
**12** Return the patient's bed to its original position, dispose of or organize materials, remove gloves, and disinfect hands.

Please drag the numbers (1–12) into the order in which you would perform these steps in practice (steps 1 to 12).

1	2	3	4	5	6	7	8	9	10
11	12								

step 1	step 2	step 3	step 4	step 5	step 6	step 7	step 8	step 9	step 10	step 11	step 12

Multimedia Appendix 4. Procedural knowledge test.

The correct order is as follows:

1. Present the patient case in front of the patient’s room, disinfect hands.

2. Greet the patient, introduce yourself, and state the reason for your visit.

3. Ask about the current pain level, adjust pain therapy if necessary, disinfect hands, and put on gown, mask, and gloves.

4. Disinfect work surfaces, set up the hygienic triangle, change gloves, and adjust the bed to working height.

5. Reposition the patient, place an absorbent underpad, and remove the dressing.

6. Assess and dispose of the old dressing, assess the wound, and change gloves.

7. Choose the wound cleansing method and product, perform wound cleansing, dispose of gloves, and disinfect hands.

8. Document the wound by taking a photo and reassess it.

9. Select wound edge protection and dressing, disinfect hands, put on gloves, and dress the wound.

10. Return the patient’s bed to its original position, dispose of or organize materials, remove gloves, and disinfect hands.

11. Educate the patient on self-care of the wound, clarify any questions, and take your leave.

12. Reflect on the wound care performed, plan next steps, and involve other professional groups if necessary.
